# A bioinformatics approach to characterize a hypothetical protein Q6S8D9_SARS of SARS-CoV

**DOI:** 10.5808/gi.22021

**Published:** 2023-03-31

**Authors:** Md Foyzur Rahman, Rubait Hasan, Mohammad Shahangir Biswas, Jamiatul Husna Shathi, Md Faruk Hossain, Aoulia Yeasmin, Mohammad Zakerin Abedin, Md Tofazzal Hossain

**Affiliations:** 1 Department of Biochemistry and Biotechnology, School of Biomedical Science, Khwaja Yunus Ali University, Sirajganj 6751, Bangladesh; 2 Department of Botany, Sirajganj Govt. College, Sirajganj 6700, Bangladesh; 3 Department of Microbiology, School of Biomedical Science, Khwaja Yunus Ali University, Sirajganj 6751, Bangladesh; 4 Department of Biochemistry and Molecular Biology, Faculty of Science, University of Rajshahi, Rajshahi 6205, Bangladesh

**Keywords:** bioinformatics, functional annotation, hypothetical protein, SARS-CoV

## Abstract

Characterization as well as prediction of the secondary and tertiary structure of hypothetical proteins from their amino acid sequences uploaded in databases by *in silico* approach are the critical issues in computational biology. Severe acute respiratory syndrome–associated coronavirus (SARS-CoV), which is responsible for pneumonia alike diseases, possesses a wide range of proteins of which many are still uncharacterized. The current study was conducted to reveal the physicochemical characteristics and structures of an uncharacterized protein Q6S8D9_SARS of SARS-CoV. Following the common flowchart of characterizing a hypothetical protein, several sophisticated computerized tools e.g., ExPASy Protparam, CD Search, SOPMA, PSIPRED, HHpred, etc. were employed to discover the functions and structures of Q6S8D9_SARS. After delineating the secondary and tertiary structures of the protein, some quality evaluating tools e.g., PROCHECK, ProSA-web etc. were performed to assess the structures and later the active site was identified also by CASTp v.3.0. The protein contains more negatively charged residues than positively charged residues and a high aliphatic index value which make the protein more stable. The 2D and 3D structures modeled by several bioinformatics tools ensured that the proteins had domain in it which indicated it was functional protein having the ability to trouble host antiviral inflammatory cytokine and interferon production pathways. Moreover, active site was found in the protein where ligand could bind. The study was aimed to unveil the features and structures of an uncharacterized protein of SARS-CoV which can be a therapeutic target for development of vaccines against the virus. Further research are needed to accomplish the task.

## Introduction

As the world is facing an outbreak of coronavirus disease 2019 caused by severe acute respiratory syndrome (SARS)–associated coronavirus 2 (SARS-CoV-2) for more than 2 years causing deaths of about six million and many more millions of infected cases [1-3], SARS has again drawn the core attention of researchers around the globe to it [4]. After its outbreak in 2003 [5,6], SARS-CoV rapidly spread into countries of the world infecting thousands of people with pneumonia-like symptoms such as dyspnea, cough, chest pain etc. [7]. SARS-infected people experience diffuse alveolar damage which might also additionally cause acute breathing misery syndrome and death [8]. To provide special support and to contain the outbreak, the World Health Organization (WHO) coordinated with the Global Outbreak Alert and Response Network (GOARN) and aided the health authorities of the SARS-affected countries [9]. SARS-CoV is an enveloped ssRNA virus [10,11] which, when enters the host (e.g., human [12], bats [13]) cell by forming a bond with a distinct enzyme angiotensin-converting enzyme 2 [14], infects the epithelial cells of the lungs [15], causing the symptoms claimed earlier. The incubation period for the virus is normally 2–7 days, but can extend to 10 days [16,17]. It is an airborne virus that can be spread by small droplets of saliva in the same means as the common cold and flu do [18,19]. SARS was the first ever severe new communicable disease emerged at the beginning of the 21st century [20] which showed a strong ability to spread by international air transport systems [5,16]. Alongside, it can also be transmitted person-to-person directly by touching each other or indirectly through infected surfaces [21,22]. Most patients previously diagnosed with SARS are healthy adults aged between 25 and 70, whereas in case of children, according to several reports, the age was limited to 15 [23,24]. According to the WHO, the mortality rate in people with the disease that was approximately 3% [25].

Proteins perform a wide range of functions within organisms, including the structure of cells and organisms, and also participate in a variety of important processes in vivo through interactions with other molecules. Millions of proteins are still uncharacterized and therefore, unveiling the biological functions and characteristics of these uncharacterized proteins of different organisms is now a common practice in the fields of bioinformatics [26-28]. SARS-CoV has a number of functional proteins [29,30], of which many are still unknown or poorly understood [31,32]. Advances in computer biology have created a variety of platforms and methods for predicting protein structure, binding sites, and biological activity [33,34]. Protein studies using bioinformatics methods make it possible to evaluate 3D structural conformations, classify novel domains, and determine functions of the proteins [35,36]. This perfect comprehension can, moreover, provide efficient pharmacological strategies for the development of promising medications for many diseases [37]. SARS-CoV has an uncharacterized accessory protein named Q6S8D9_SARS. However, the physicochemical properties, secondary, and tertiary structures with the active ligand binding site of the protein are not yet published. Therefore, our study was intended to predict the structure and biological functions of the uncharacterized protein by using various biological information methods and tools. It is imperative to analyze the functional annotation of the uncharacterized protein as well as to increase understanding of the protein as a possible drug target.

## Methods

### Selection of the hypothetical protein for characterization

Hypothetical proteins were found in the NCBI (https://www.ncbi.nlm.nih.gov) [38] protein database while searching using the term "hypothetical protein of SARS-CoV" and the resulting hits were picked at random to investigate the near relatives using BLAST programs. To anticipate the protein's function, a resemblance search was conducted using NCBI power tools to identify proteins with functional and structural similarities to the hypothesized protein.

### Sequence retrieval

With the Taxonomy ID 258507, the amino acid sequence in FASTA format of Q6S8D9_SARS protein was retrieved from the NCBI database and then saved. Q6S8D9 was found as ‘uncharacterized protein’ in the Protein Data Bank (PDB) (https://www.rcsb.org), since its function and structures hadn't been discovered yet.

### Physicochemical properties analysis

For the assessment of the physical and chemical properties of the uncharacterized protein, we used the ExPASy Protparam tool (https://web.expasy.org/protparam) [39].

### Functional annotation prediction

Domain prediction was done using NCBI’s CD Search tool (https://www.ncbi.nlm.nih.gov/Structure/cdd/wrpsb.cgi) [40].

### Secondary structure modeling

The amino acid FASTA sequence was utilized to retrieve the secondary structure elements of the hypothetical protein employing the SOPMA server [41] and the PSIPRED tool (http://bioinf.cs.ucl.ac.uk/psipred/) [42].

### Tertiary structure modeling and validation

In the PDB, we found no experimentally determined 3D structure for Q6S8D9_SARS. As a result, three separate programs, Modeller [43] with the HHpred tool [44], the Phyre2 [45], and the Swiss-Model server [46], were used to model the protein's tertiary structures. Then, the structural quality of anticipated tertiary structures derived from the tools was tested. The Ramachandran plot analysis by PROCHECK [46], and the Swiss-Model Interactive Workspace (https://swissmodel.expasy.org/assess) [47] were utilized to document the quality and feature of the modeled structure. Z-scores produced from the Swiss-Model server and bond angles from the ProSA-web (https://prosa.services.cam.sbg.ac.at/prosa.php) server [48] also required for the consistency evaluation of the entire model.

### Active site prediction

We used the CASTp v.3.0 server [49] to find, delineate, and measure the active site of the uncharacterized protein. Basically, the CASTp server uses a test sweep and protein structures from the PDB as input for topographic computing. In addition, the CASTp server provides topographic features. The outcomes can be easily downloaded from the server and seen using PymoL [50].

### Accession number

The accession numbers for the protein sequence reported in this paper are [UniProt database]: Q6S8D9 (primary or citable), J9TE29 (secondary).

## Results and Discussion

**Fig. 1.**
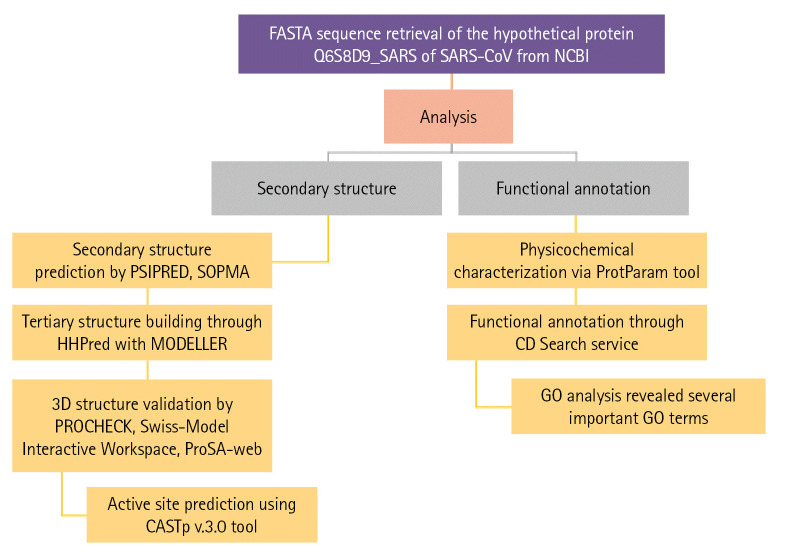
Flow chart of the proposed study. GO, gene ontology; SARS-CoV, severe acute respiratory syndrome–associated coronavirus.

The complete workflow of our study has shown in Fig. 1.

### Physicochemical characteristics of the uncharacterized protein

**Fig. 2.**
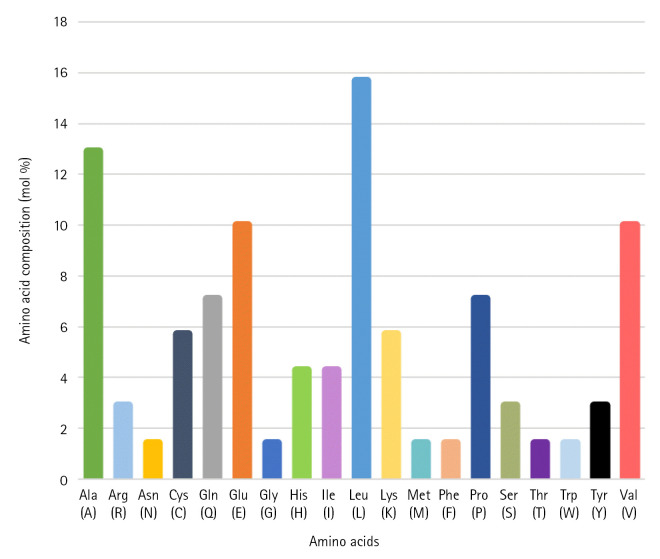
Amino acid composition of the hypothetical protein Q6S8D9_SARS. SARS, severe acute respiratory syndrome.

The FASTA format sequence of the Q6S8D9 protein of SARS-CoV was used to assess the physicochemical parameters [51]. The hypothetical protein consists of 70 amino acids and has a total molecular weight of 7,852.33 Da. The theoretical pI was calculated to be 6.25 and the protein's molecular formula was determined to be C_356_H_573_N_93_O_96_S_5_. In addition, the overall positively (Arg + Lys) and negatively (Asp + Glu) charged residues were 6 and 7 in numbers, respectively. The presence of Cys, Trp, and Tyr residues is indicated by a high Extinction coefficient of 8,730. The query protein has a higher aliphatic index value of 119.86, indicating that it is stable over a wide temperature range [52]. Because its instability index (26.67) is less than 40, the protein remains unchanged in nature which represents stability [53]. Because of the positive higher grand average of hydropathicity (GRAVY) indices value of 0.310, the protein has polarity [54]. Table 1 displays all of the physicochemical property results which will help to identify drug or vaccine target while Fig. 2 shows the amino acid composition.

### Functional annotation prediction and gene ontology analysis

A domain is a specific part of a protein sequence which acts as the structural and functional basis of the protein [55]. A domain named SARS-CoV_ORF9c superfamily (accession ID: cl38891) was found by the CD Search tool which may trouble host antiviral inflammatory cytokine and interferon production pathways [56]. On the other hand, gene ontology (GO) analysis was performed via Predict Protein tool [57] to interpret the biological activities of the protein and to underscore the most relevant GO terms associated with the protein. Table 2 represents all three categorized GO terms with their reliability values.

Changes in the biological processes were mostly enriched in locomotion, viral release from host cell, multi-organism process, viral DNA genome packaging, and obsolete movement other organisms. Significant alteration in the cellular component was found in the host cell nucleus. In addition, alterations in the molecular functions were significantly related with DNA-binding transcription factor (TF) activity, RNA polymerase II TF binding, and bHLH TF binding.

### Secondary structure analysis

**Fig. 3.**
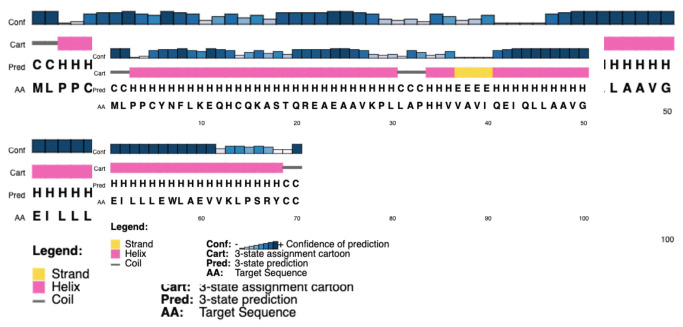
Secondary structure of the hypothetical protein developed by PSIPRED.

To demonstrate the secondary structure, the SOPMA tool was employed with its default settings which produced periodic proportions of alpha helix, beta-turn, extended strand, and random coil of protein of 81.43%, 1.43%, 1.43%, and 15.71%, respectively (Table 3). PSIPRED predicted the helix, strand, and coil with a higher level of certainty (Fig. 3).

### Tertiary structure analysis and validation

**Fig. 4.**
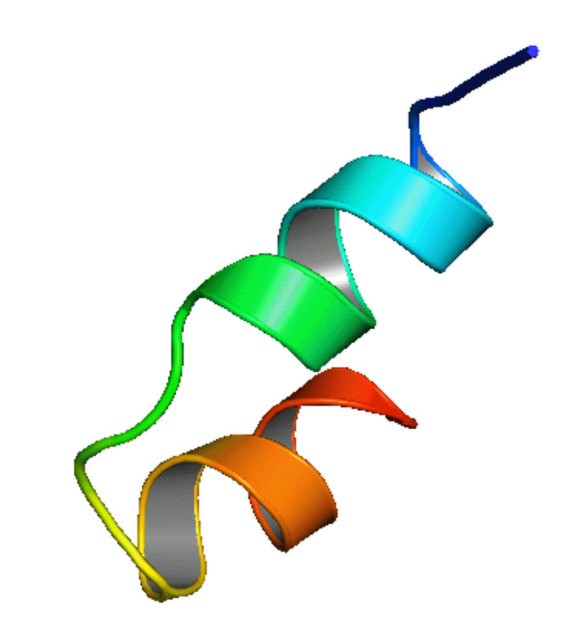
Tertiary structure of Q6S8D9_SARS protein predicted by HHpred with Modeller tool. SARS, severe acute respiratory syndrome.

We employed three sophisticated bioinformatics tools, the HHpred with Modeller, the Phyre2, and the Swiss-Model server, to construct the 3D structure of Q6S8D9_SARS protein. After uploading the query amino acid sequence in HHpred’s [44] input box, the tertiary structure was developed by selecting the most appropriate template 1FVY A, which featured the highest probability rate (33.71%), the E-value of 97, score of 16.7, an SS of 3.1, Aligned Cols of 25 and a target length of 31 (data not shown), of the 11 hits. 1FVY A is the solution structure of the human parathyroid hormone's osteogenic 1–31 fragment [58]. The modeled tertiary structure of the Q6S8D9 protein was then saved in a PDB format and afterward viewed in Modeller. Likewise, the Phyre2 tool [45] was also used for the prediction tertiary structure where the template (b6e5oD) was chosen depending on the following two factors: confidence value (100%) and coverage (98.7%). Furthermore, we employed the Swiss-Model tool [46] also to construct the 3D structure of Q6S8D9_SARS protein by reckoning the most probable template (6b4e.1.A) that shows the values of GMQE and QMEANDisCo Global of 0.34 and 0.41, respectively and covers 18.37% sequence identity with Nucleoporin GLE1 protein. All the tools that were employed to develop the tertiary structure gave the same 3D structures of the hypothetical protein. Fig. 4 shows the 3D structure of the protein which was constructed using the HHpred tool and shown by the Modeller.

**Fig. 5.**
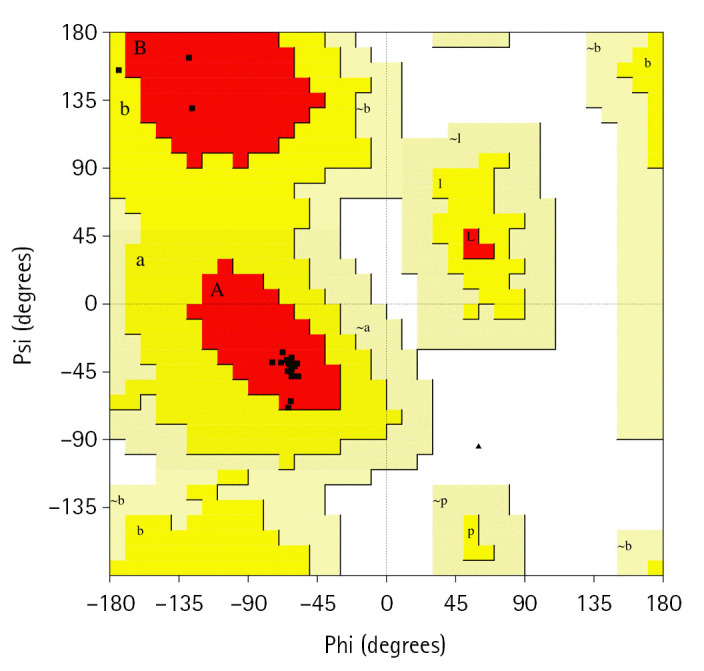
Ramachandran plot of the hypothetical protein.

After constructing the tertiary structure, we employed another two bioinformatics tools, PROCHECK and the Swiss-Model Interactive Workspace, to assess the validity of the obtained structure. The PDB file of the tertiary structure of the protein was uploaded and then run in the PROCHECK tool which resulted in the Ramachandran plot and other features. The Ramachandran plot statistics (Fig. 5) showed that a number of 21 residues (95.5%) was found in the most favored regions whereas 4.5% of total residues were in the additional allowed regions [a,b,l,p]. However, no residue was uncovered in the generously allowed and disallowed areas. In addition, among the total residues, the non-glycine and non-proline residues, end-residues excluding glycine and proline, and glycine residues valued 100%, 2%, and 1%, respectively (Table 4).

On the other hand, the Ramachandran plot constructed by the Phyre2 and Swiss-Model servers resulted that, of the total residues 94.3% and 95.1% were found in the [A, B, L] areas, respectively, which validate our obtained tertiary structure. In addition, 6.5% and 5.9% residues were pitched in the additional allowed regions and 0.4% and 0.2% were found in the disallowed regions, respective of the servers. However, no residue was found in the generously allowed regions in the Phyre2 tool (Table 4).

In case of the Swiss-Model Interactive Workspace, another validating tool, 93.88% residues designated as the Ramachandran favored and the MolProbity Score calculated to be 1.82 which also positively evaluate the 3D structure of the hypothetical protein. Among the other features of the Swiss-Model Interactive Workplace, Z-scores of the QMEAN (Qualitative Model Energy Analysis), Cβ, all atom pairwise, solvation energy, and the torsion angle value were found −1.76, −1.68, −0.78, −0.80 and −1.32, respectively, which also supported the proteins’ tertiary structure (Table 5). Furthermore, the 3D structures of the Q6S8D9_SARS protein were confirmed by the ProSA-web [48] server by determining the standard bond angles and degree of nativeness of the hypothetical protein.

### Active site of the hypothetical protein

**Fig. 6.**
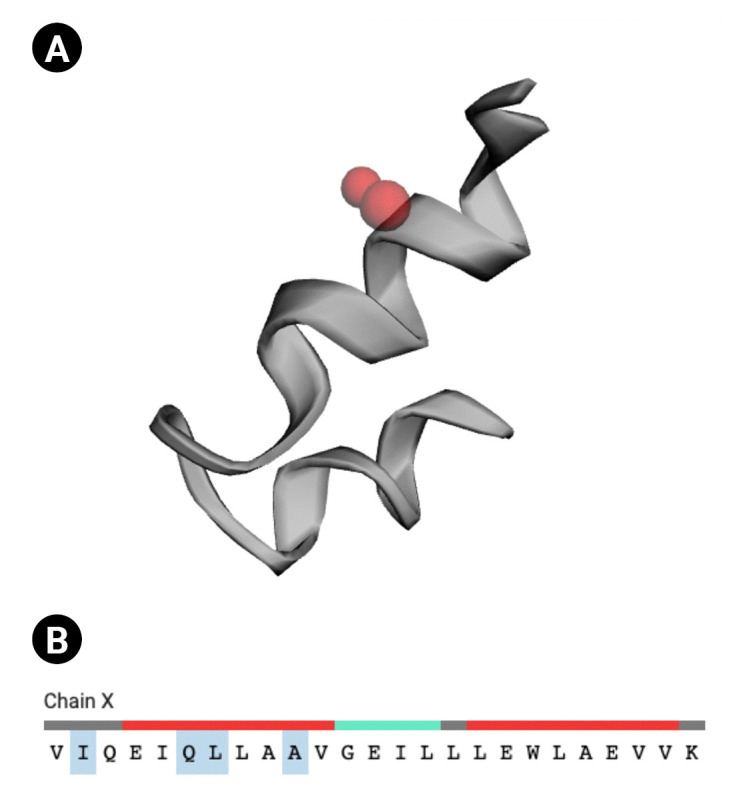
Active site of the Q6S8D9 protein. (A) Red sphere denoting the active sites. (B) Four amino acid residues (ILE, GLN, LEU, and ALA) in the active site (shaded).

CASTp v.3.0 [49], a sophisticated server for locating surface pockets of a protein, was executed to locate the functional site of the Q6S8D9 protein. We found that, among the 70 amino acid residues, only four residues (Sequence ID: 40, 44, 45, and 48) act as active site (red sphere in Fig. 6A and 6B) for the protein. The active site possesses an area of 2.144 and a volume of 0.108.

Characterization of a protein using sophisticated bioinformatics tools is another novel task as like as other systems biology works. In our study, we aimed to reveal the physicochemical characteristics, structures and functions of a hypothetical protein Q6S8D9_SARS of SARS-CoV. The 70 amino acid containing protein contains more negatively charged residues and a high aliphatic index value and a low instability index value make the protein more temperature stable. The secondary structure modeled by several bioinformatics tools ensured that the proteins had domain in it which indicated it was a functional protein and tertiary structure prediction showed the protein had a fine 3D structure validated by various servers. Moreover, active site was found in the protein where ligand could bind. Further study of the protein is needed to find novel therapeutic drug for the SARS-CoV treatment targeting the protein.

## Figures and Tables

**Table 1.** Physicochemical characteristics of the Q6S8D9_SARS protein

Properties	Value
No. of amino acids	70
Molecular weight	7,852.33
Theoretical pI	6.25
Total number of negatively charged residues (Asp + Glu)	7
Total number of positively charged residues (Arg + Lys)	6
Total number of atoms	1,123
Extinction Coefficient (all pairs of Cys residues form cysteines)	8,730
Extinction Coefficient (all Cys residues are reduced)	8,480
Half-life (*in vitro*) (h)	30
Instability index (II)	26.67
Aliphatic index	119.86
Grand average of hydropathicity	0.31

**Table 2.** Predicted functions of the hypothetical protein

Category	GO ID	GO term	Reliability (%)
Biological process	GO:0040011	Locomotion	37
	GO:0019076	Viral release from host cell	37
	GO:0051704	Multi-organism process	37
	GO:0019073	Viral DNA genome packaging	37
	GO:0052192	Obsolete movement in environment of other organism involved in symbiotic interaction	37
Cellular component	GO:0042025	Host cell nucleus	37
Molecular function	GO:0003700	DNA-binding transcription factor activity	35
	GO:0046983	Protein dimerization activity	35
	GO:0001085	RNA polymerase II transcription factor binding	35
	GO:0043425	bHLH transcription factor binding	35

GO, gene ontology.

**Table 3.** Secondary structure element of the uncharacterized protein

Secondary structure elements	Value (%)
Alpha helix	81.43
3_10_ helix (Gg)	0
Pi helix (Ii)	0
Beta bridge (Bb)	0
Extended strand (Ee)	1.43
Beta turn (Tt)	1.43
Bend region (Ss)	0
Random coil (Cc)	15.71
Ambiguous states	0
Other states	0

**Table 4.** Ramachandran plot statistics of the hypothetical protein

Tools	Ramachandran plot statistics	Value (%)
PROCHECK	Residues in the most favored regions [A, B, L]	95.5
	Residues in the additional allowed regions [a, b, l, p]	4.5
	Residues in the generously allowed regions [~a, ~b, ~l, ~p]	0
	Residues in the disallowed regions	0
	Number of non-glycine and non-proline residues	1
	Number of end-residues (excl. Gly and Pro)	2
	Number of glycine residues (shown in triangles)	1
	Number of proline residues	0
	Total number of residues	25
Phyre2	Residues in the most favored regions [A, B, L]	94.3
	Residues in the additional allowed regions [a, b, l, p]	6.5
	Residues in the generously allowed regions [~a, ~b, ~l, ~p]	0
	Residues in the disallowed regions	0.4
Swiss-Model	Residues in the most favored regions [A, B, L]	95.1
	Residues in the additional allowed regions [a, b, l, p]	5.9
	Residues in the generously allowed regions [~a, ~b, ~l, ~p]	0.3
	Residues in the disallowed regions	0.2

**Table 5.** Z-scores of scoring function terms in Swiss-Model server

Scoring function term	Z-score
QMEAN score	–1.76
C_b interaction energy	–1.68
All atom pairwise energy	–0.78
Solvation energy	–0.80
Torsion angle energy	–1.3
